# The role of pituitary adenylate cyclase-activating polypeptide neurons in the hypothalamic ventromedial nucleus and the cognate PAC1 receptor in the regulation of hedonic feeding

**DOI:** 10.3389/fnut.2024.1437526

**Published:** 2024-08-21

**Authors:** Sarah Sayers, Nikki Le, Edward J. Wagner

**Affiliations:** College of Osteopathic Medicine of the Pacific, Basic Medical Sciences, Western University of Health Sciences, Pomona, CA, United States

**Keywords:** pituitary adenylate cyclase-activating polypeptide, hypothalamic ventromedial nucleus, ventral tegmental area, A_10_ dopamine neurons, food addiction, PAC1 receptor, hedonic feeding

## Abstract

Obesity is a health malady that affects mental, physical, and social health. Pathology includes chronic imbalance between energy intake and expenditure, likely facilitated by dysregulation of the mesolimbic dopamine (DA) pathway. We explored the role of pituitary adenylate cyclase-activating polypeptide (PACAP) neurons in the hypothalamic ventromedial nucleus (VMN) and the PACAP-selective (PAC1) receptor in regulating hedonic feeding. We hypothesized that VMN PACAP neurons would inhibit reward-encoding mesolimbic (A10) dopamine neurons via PAC1 receptor activation and thereby suppress impulsive consumption brought on by intermittent exposure to highly palatable food. Visualized whole-*cell* patch clamp recordings coupled with *in vivo* behavioral experiments were utilized in wildtype, PACAP-*cre*, TH-*cre*, and TH-*cre*/PAC1 receptor-floxed mice. We found that bath application of PACAP directly inhibited preidentified A_10_ dopamine neurons in the ventral tegmental area (VTA) from TH-*cre* mice. This inhibitory action was abrogated by the selective knockdown of the PAC1 receptor in A_10_ dopamine neurons. PACAP delivered directly into the VTA decreases binge feeding accompanied by reduced meal size and duration in TH-*cre* mice. These effects are negated by PAC1 receptor knockdown in A_10_ dopamine neurons. Additionally, apoptotic ablation of VMN PACAP neurons increased binge consumption in both lean and obese, male and female PACAP-*cre* mice relative to wildtype controls. These findings demonstrate that VMN PACAP neurons blunt impulsive, binge feeding behavior by activating PAC1 receptors to inhibit A_10_ dopamine neurons. As such, they impart impactful insight into potential treatment strategies for conditions such as obesity and food addiction.

## 1 Introduction

Obesity is a health malady that affects an individual’s mental, physical, and social health (1). Furthermore, obesity promotes the development of metabolic and physiological comorbidities such as, but not limited to, type 2 diabetes, hypertension, cardiovascular disease, obstructive sleep apnea, and respiratory disease (2, 3). Pathology of obesity includes prolonged imbalance between energy expenditure and energy intake (4). The cause of this imbalance is multifactorial, however, a main proponent may stem from the hedonic control of appetite and food intake. The hedonic pathway utilizes food reward cues to modulate energy intake and expenditure (5). Rodent studies investigating motivated behavior suggest that substance abuse and consumption of palatable foods converge at the same pathway within the limbic system (5–7). This pathway is comprised of dopaminergic (DA) signaling from A_10_ dopamine neurons originating at the ventral tegmental area (VTA) of the mesencephalon and terminating in the nucleus accumbens (NAc) (8). These neurons comprise the mesolimbic pathway (9) and are associated with regulation of behaviors motivated by reward (10). The release of DA in the NAc has been shown to facilitate goal directed motor behavior (11). In relation to obesity, an individual’s consistent consumption of highly palatable foods over-activates this dopaminergic circuitry as it becomes habitually stimulated. This repeated activation of the mesolimbic pathway dampens the self-regulation of the mesolimbic circuitry, thereby rendering the subject susceptible to compulsive actions such as overeating or binge eating behaviors (12, 13).

Previous studies have depicted pituitary adenylate cyclase-activating polypeptide (PACAP) as a prominent regulator of feeding behavior (14–16). PACAP is abundantly expressed in the hypothalamic ventromedial (VMN), paraventricular (PVN), and arcuate (ARC) nuclei (17–19), which are implicated in homeostatic regulation of feeding. Administration of PACAP into the ARC, VMN, and PVN has been evidenced to decrease food intake and increase metabolic parameters such as locomotor activity, core body temperature, and O2 consumption (20–23). There are two populations of appetite-regulating PACAP neurons within the hypothalamus: one in the VMN and the other in the PVN. VMN PACAP neurons co-express steroidogenic factor-1 and glutamate, and are considered anorexigenic (18, 22, 24). PVN PACAP neurons co-express thyrotropin-releasing hormone and glutamate, and are considered orexigenic (19, 25). There are also two classes of receptors to which PACAP can bind. The PACAP-specific PAC1 receptor is highly selective for PACAP, whereas the VPAC1 and VPAC2 receptors have comparable affinity for both PACAP and vasoactive intestinal polypeptide (26). The PAC1R system is highly expressed within the dorsomedial nucleus (DMN), PVN, ARC, and VMN of the hypothalamus (15, 26, 27).

Activation of the PAC1R in anorexigenic proopiomelanocortin (POMC) neurons within the ARC elicits Gq-and phosphatidylinositol-3-kinase (PI3K)-mediated signaling that links this cognate receptor to transient receptor potential channel 5 (TRPC5) channels (22, 23). Opening of these channels upon PAC1R activation in POMC neurons leads to calcium and sodium influx and consequent depolarization of these cells, an effect that is potentiated by estradiol in females (22, 28). On the other hand, orexigenic ARC NPY/AgRP neurons express PAC1R and VPAC2R (29). PVN PACAP neurons provide excitatory input to ARC NPY/AgRP neurons, and are therefore able to modulate consumption via these orexigenic neurons (19). However, in keeping with their prominent anorexigenic role, optogenetic stimulation of VMN PACAP neurons, as well as bath application of exogenous PACAP powerfully hyperpolarizes NPY/AgRP neurons via Gq-coupled, PAC1R-mediated activation of K_ATP_ channels (23).

Investigation of the effects of PACAP in both the homeostatic and hedonic circuitries reveal PACAP exerts pleiotropic effects. While PACAP elicits excitation of ARC POMC neurons under *ad libitum*-fed conditions, deviations from homeostasis have been shown to either attenuate this response (in the case of diet-induced obesity), or reverse its polarity altogether (in the case of fasting) due to a switch in the PAC1R coupling from TRPC5 channels to K_ATP_ channels, which promote potassium efflux and hyperpolarization of the postsynaptic neuron (22, 30). We have also shown that the effect of PACAP on NPY/AgRP neurons reverses polarity from predominantly inhibitory to mostly excitatory under conditions of fasting (23). Injections of PACAP into the NAc reduces hedonic feeding and drive (31, 32). Additionally, PACAP and its cognate PAC1R are expressed in the NAc and VTA, which are evidenced to be involved in the hedonic regulation of feeding (33, 34). Similarly, intra-VTA PACAP administration suppresses the binge-like consumption of palatable food in lean mice due to activation of K_ATP_ channels and hyperpolarization of A_10_ DA neurons in the VTA (35), which is blocked by the PAC1/VPAC2R antagonist PACAP_6-38_. A similar polarity switch occurs in A_10_ dopamine neurons from obese, high fat diet (HFD)-fed females, where the PACAP response flips from predominantly inhibitory to excitatory (5).

Seeing as the VMN PACAP neurons project to and synapse with VTA neurons, and with supporting evidence from previous studies depicting decreased drive for palatable food upon administration of PACAP to the VTA (35), it is hypothesized that selective knockdown of the PAC1R in A_10_ DA neurons will diminish the activation of K_ATP_ channels and subsequent inhibition of these cells; thereby blunting the PACAP-induced suppression of binge feeding. Furthermore, we postulate apoptotic ablation of VMN PACAP neurons will enhance the hedonic drive for highly palatable food evidenced by increased consumption during intermittent exposure to HFD. This research brings further clarity to the hedonic energy balance circuitry and the mechanisms driving food addiction and obesity, as well as potential therapeutic targets to alleviate hedonic drive and promote a return to homeostasis between energy intake and expenditure.

## 2 Materials and methods

### 2.1 Animal models

All animal care and procedures were compliant with Western University of Health Sciences’ Institutional Animal Care and Use Committee, and the NIH Guide for the Care and Use of Laboratory Animals. The presented study utilizes PACAP-*cre* and tyrosine hydroxylase (TH-*cre*) transgenic mice populations purchased from Jackson Laboratories (Stock #030155, #008601 respectively, Bar Harbor, ME, United States). These strains were generated on a C57BL/6 background and bred in house. PAC1R^fl/fl^ mice were obtained from Dr. Rachel Ross (Albert Einstein College of Medicine, Bronx, NY, United States), and bred with TH-*cre* mice to produce double transgenic TH-*cre*/PAC1R^fl/fl^ mice (for experimentation lean mice: 16–25 g, 12–26 weeks; HFD mice: 18–35 g, 14–30 weeks). Additionally, wildtype mice bred in house on a C57BL/6 background were utilized. A total of 175 males and 41 ovariectomized females were utilized. Animals were provided food and water *ad libitum*, kept on a 12 h light–12 h dark schedule (light 06:00–18:00), and maintained under 25°C. At 21 days of age, pups were weaned and genotyped utilizing standard PCR protocols. Animals were subsequently assigned to either a standard chow diet (Teklad Rodent Diet, Teklad Diets, Madison, WI, United States) with 18% of calories derived from fat, 24% from protein, and 58% from carbohydrates, or they were assigned to a high fat diet (HFD) group which derived 45% of calories from fat, 20% from protein, and 35% from carbohydrates 5–8 weeks prior to experimentation.

### 2.2 Surgical procedures

Approximately 5 days prior to experimentation, all female TH-*cre* and TH-*cre/*PAC1R^fl/fl^ mice were ovariectomized (OVX) under 2% isoflurane anesthesia. Ovariectomies were utilized to control the estrous cycle and study sex differences. Surgical outfitting with a 26-gauge guide cannula (Plastics One, Roanoke, VA, United States) or stereotaxic injection of adeno-associated viral vector constructs (AAV) was performed in TH-*cre*, TH-*cre/*PAC1R^fl/fl^, PACAP-*cre*, and wildtype mice. Animals were placed in a stereotaxic frame (Stoelting, Wood Dale, IL, United States), and anesthetized with 2% isoflurane. An incision was made to expose the skull, and either a unilateral or bilateral holes were drilled on one or both sides of the mid-sagittal suture to allow the guide cannula or injection needle to be lowered into the VTA (from bregma AP: −2.1 mm; ML: ±0.5 mm; DV: −4.0 mm from dura) or VMN (from bregma AP: −0.6 mm; ML: ±0.3 mm; D: −5.6 mm from dura). Injections of *cre-recombinase* dependent AAV1 containing either enhanced yellow fluorescent protein (eYFP) blank control (pAAV-Ef1a-DIO EYFP; 1.0 × 10^13^, 300 nL total volume, Addgene, plasmid #27056, deposited by Karl Deisseroth), or caspase-3 (pAAV-flex-taCasp3-TEVp; 7 × 10^12^ genomic copies/mL); 300 nL total volume (gift from Nirao Shah and Jim Wells; Addgene plasmid #45580). Animals were used 2–4 weeks following AAV eYFP injection/surgical implantation with guide cannula for experimentation. Only animals with correct placement were included in experimentation data. Wildtype and PACAP-*cre* animals who received bilateral VMN injections with AAV caspase-3 were utilized for experimentation 4 weeks post injection to allow adequate time for viral transfection and subsequent VMN PACAP specific ablation to occur.

### 2.3 Drugs

All drugs were purchased from Tocris Bioscience/ R&D Systems (Minneapolis, MN, United States), unless stated otherwise. For electrophysiological experiments, the PAC1R agonist, PACAP_1-38_ was prepared as a 100 μM stock solution in UltraPure H_2_O and further diluted with artificial cerebrospinal fluid (aCSF) to a working concentration of 100 nM. For behavioral experiments, the PAC1/VPAC2 antagonist, PACAP_6–38_ was prepared as a 200 mM stock solution, and PACAP_1–38_ was prepared as a 150 μM stock, and these two stock solutions were further diluted to 1 nM and 30 pM solutions, respectively, by dissolving them in filtered saline.

### 2.4 Midbrain slice preparation

On experiment day, 32% isoflurane was utilized to briefly anesthetize the animal (TH-*cre* or TH-*cre/*PAC1R^fl/fl^) prior to rapid decapitation. The brain was carefully and swiftly extracted from the skull and a coronal mesencephalic block was procured. The block was then mounted on a cutting platform and secured in a vibratome filled with ice-cold, oxygenated, sucrose-based cutting solution (NaHCO_3_ 26; dextrose 10, HEPES 10; sucrose 208; KCl 2; NaH_2_PO_4_ 1.25; MgSO_4_ 22; CaCl_2_ 1; in mM). Two to three slices at 250 um were obtained through the rostrocaudal aspect of the VTA. The slices were then transferred to an auxiliary chamber containing room temperature oxygenated aCSF containing the following (mM):

NaCl, 124; NaHCO3 26; dextrose 10, HEPES 10; KCl 5; NaH2PO4 2.6; MgSO4 2; CaCl2 1

### 2.5 Electrophysiology

Whole-cell patch clamp electrophysiological recordings from VTA neurons were performed utilizing biocytin-filled electrodes. During recordings, the slices were maintained in a chamber under continuous perfusion with oxygenated aCSF (35°C), with the CaCl_2_ concentration raised to 2 mM. Artificial CSF and all drugs diluted with aCSF were perfused via a peristaltic pump at a rate of 1.5 mL/min. Borosilicate glass (World Precision Instruments, Sarasota, FL, United States; 1.5 mm OD) patch electrodes were pulled on a P-97 Flaming Brown Puller (Sutter Instrument Co., Novato, CA, United States), and filled with an internal solution containing the following: Potassium gluconate 128; NaCl 10; MgCl2 1; EGTA 11; HEPES 10; ATP 1; GTP 0.25; (in mM) and 0.5% biocytin. Internal solution was adjusted to a pH of 7.3 with KOH; osmolality: 286–320 mOsm. Recording electrode resistances ranged from 3 to 8 MΩ.

Recordings were visualized using an Olympus BX51 W1 fixed stage microscope outfitted with infrared differential interference contrast (DIC) video imaging. Multiclamp 700A or B preamplifiers (Molecular Devices) amplified potentials and passed current through the electrode. Analog-digital conversion of membrane current was carried out using Digidata 1550A or B interfaces (Molecular Devices) coupled to pClamp 10.6 or 11.0 software. Access resistance, resting membrane potential (RMP), and input resistance were monitored for the entirety of recordings. Recording was ended if access resistance deviated more than 10% of the original value. Low-pass filtering of the currents was conducted at a frequency of 2 kHz. Liquid junction potential was calculated as −10 mV, and corrected during data analysis with pClamp software. Recordings were performed at holding potential of −60 mV.

Initial baseline current–voltage (I/V) relationship was generated in slices from TH-*cre* or TH-*cre* PAC1R^fl/fl^ mice injected 2–3 weeks prior with eYFP blank AAV into the VTA using a ramp protocol (75 mV/s; from −110 to −30 mV). Following baseline I/V, PACAP_1-38_ [100 nM] was bath applied and the membrane current was continuously monitored until a new steady-state value was observed. Following this, a second I/V relationship was recorded. Membrane current was again continuously monitored as PACAP washed out and a final I/V relationship was recorded.

### 2.6 Behavioral studies

Behavioral studies were conducted utilizing a four station Comprehensive Lab Animal Monitoring System (CLAMS; Columbus Instruments, Columbus, Ohio, United States) as previously described and validated (36). Meal pattern and energy intake in intact male and OVX female wildtype, PACAP-*cre*, TH-*cre*, and TH-*cre*/PAC1R^fl/fl^ mice were monitored. Prior to executing our binge feeding protocol, animals were allowed to acclimate in the CLAMS chambers for 3 days where they were handled, weighed, and returned to their cages every afternoon. Following acclimation, binge feeding was monitored over the course of 5 consecutive days as previously described (37, 38). Briefly, animals were exposed to HFD from 16:00 to 17:00, and at the end of each 1-h exposure, they were switched back to standard chow for the remaining 23 h. For the studies involving apoptotic ablation of VMN PACAP neurons, lean and obese male and OVX female wildtype and PACAP-*cre* animals were randomly assigned to either standard chow or HFD 5 weeks prior to experimentation. Subjects were injected with a caspase-3-containing AAV into the VMN 4 weeks prior to experimentation. Long term HFD-fed animals were switched back to standard chow a week before experimentation, then reintroduced to HFD for the binge hour as described above. For the studies involving TH-*cre* and TH-*cre*/PAC1R^fl/fl^ animals, subjects were injected with PACAP_1_-_38_ (30 pmol, 0.2 μL), PACAP_6–38_ (1 nM, 0.2 μL), or its 0.9% saline vehicle (0.2 μL) directly into the VTA just prior to the HFD exposure hour.

### 2.7 Immunohistochemistry

Slices containing the VTA region from TH-*cre* and TH-*cre*/PAC1R^fl/fl^ mice were fixed with 4% paraformaldehyde (PFM) in Sorenson’s phosphate buffer (pH 7.4) overnight. Following the fixation period, slices were then immersed for 3 days in 20% sucrose dissolved in Sorenson’s buffer which was changed daily. 2-methylbutane (EMD Millipore Corporation, Burlington, MA, United States) was utilized to snap freeze slices. Coronal sectioning (20 μM) through the VTA was conducted on a cryostat and mounted on chilled slides. Sections were then washed with 0.1 M sodium phosphate buffer (PBS; pH 7.4), and then processed overnight with a polyclonal antibody directed against PAC1R (ABCAM; Cambridge, MA, United States; AB28670; 1:500 dilution). The following day, two 15-min washes with PBS, and a 2 h incubation period with secondary biotinylated goat anti-rabbit antibody (Jackson ImmunoResearch Laboratories, Inc., West Grove, PA, United States; 1:300), then three 15 min washes with PBS and another 2 h overlay with streptavidin-Alexa Fluor 546 (Molecular Probes Inc., Eugene, OR, PA, United States 1:600) was conducted. This was followed by a final series of three 30-min washes. For TH immunolabeling, these same slides were washed in PBS as described above and processed overnight with a monoclonal antibody for TH (Immunostar, Inc., Hudson, WI, United States; 1:4,000 dilution). The following day, slides were washed with PBS twice for 15 min and secondary goat anti-mouse antibody conjugated with Alexa Fluor 488 (Life Technologies, Carlsbad, CA, United States, 1:300) was utilized for the 2 h overlay. Following the final series of three 30 min washes with PBS, slides were coverslipped and evaluated using fluorescence immunohistochemistry on a Zeiss Axioskop 2 Plus microscope (Carl Zeiss, Gӧttingen, Germany). Percent colocalization was calculated bia comparison of TH and PAC1R fluorescence. PAC1R positive and TH positive cells were divided by the total number of TH positive cells. Representative sections were evaluated in triplicate from three TH-*cre* mice and three TH-*cre*/PAC1R^fl/fl^ mice. Cell counts were taken from 0.12 mm^2^ area in the VTA and determined in triplicate (22, 23).

### 2.8 Statistical analysis

Student’s *t*-test or Mann–Whitney U-test were utilized to draw comparisons between two groups. One-way or repeated measures, multifactorial ANOVA, followed by Least Significant Difference (LSD) test were utilized for comparisons made between more than two groups. An alpha probability of <0.05 was necessary for a difference to be considered statistically significant.

## 3 Results

### 3.1 The PAC1R is effectively knocked down in TH-*cre*/PAC1R^fl/fl^ mice

**Figure 1:**
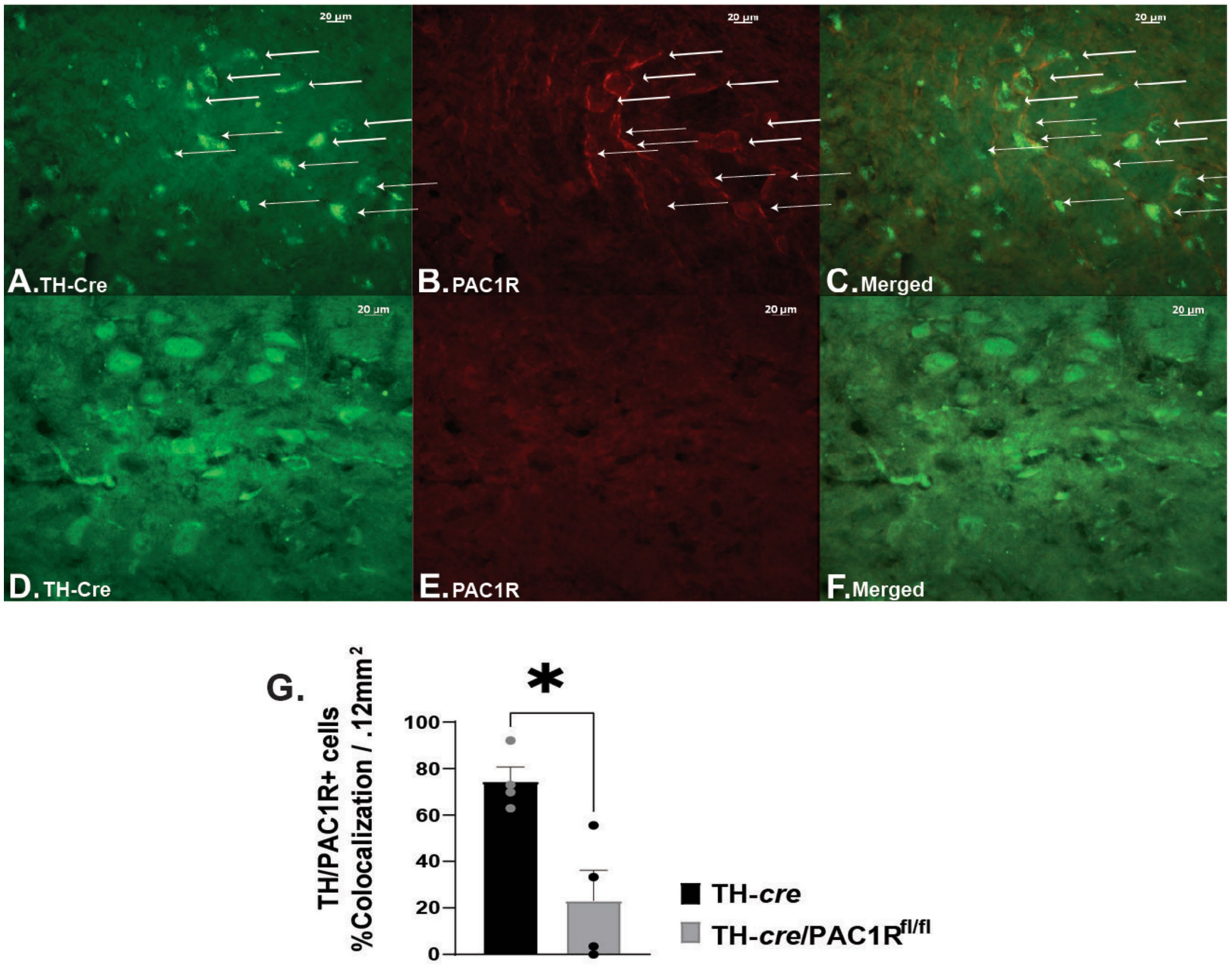
Validation of PAC1R knockdown in TH-*cre*/PAC1R^fl/fl^ mice. Photomicrographs on the left represent TH immunostaining (visualized with Alexa Fluor 488) in sections from TH-*cre* **(A)** and TH-*cre*/PAC1R^fl/fl^ **(D)** mice. Photomicrographs in the middle illustrate PAC1R immunostaining (visualized with Alexa Fluor 546) in the same sections from TH-*cre* **(B)** and TH-*cre*/ PAC1R^fl/fl^ **(E)** mice. The photomicrographs in panels **(C,F)** denote merged images depicting retention of PAC1R in A_10_ DA neurons (denoted by the arrows) from TH-*cre* but not TH-*cre*/PAC1R^fl/fl^ mice. **(G)** Percent of cells positive for colocalization of TH and PAC1R in TH-*cre* and TH-*cre*/PAC1R^fl/fl^ mice. Mann–Whitney U-test, ^*^p < 0.05, TH-*cre n* = 4, TH-*cre*/PAC1R^fl/fl^ *n* = 4. Bars represent means and lines 1 SEM.

Intra-VTA administration of PACAP_1–38_ has been associated with decreased binge-like consumption of palatable food due to K_ATP_ channel activation and hyperpolarization of VTA A_10_ DA neurons (35). This effect is blocked by the PAC1/VPAC2R antagonist PACAP_6-38_ (35). To confirm whether, indeed, the PAC1R is the mediator for the PACAP-induced hyperpolarization of A_10_ DA neurons, we utilized double transgenic TH-*cre*/PAC1R^fl/fl^ mice to determine if inhibition persisted when the PAC1R was knocked down. We first wanted to validate the knockdown of the PAC1R expression in VTA neurons. We compared coronal VTA slices from TH-*cre* (Figures 1A–C) to TH-*cre*/PAC1R^fl/fl^ mice (Figures 1D–F) that we immunostained with antibodies directed against TH and the PAC1R, and confirmed the PAC1R is appreciably knocked down in A_10_ DA neurons from TH-*cre*/PAC1R^fl/fl^ animals (Figure 1G: Mann–Whitney U-test: W = 0, *p* < 0.04).

### 3.2 PAC1R is necessary for PACAP induced VTA A_10_ DA neuron hyperpolarization in TH-*cre* mice

**Figure 2:**
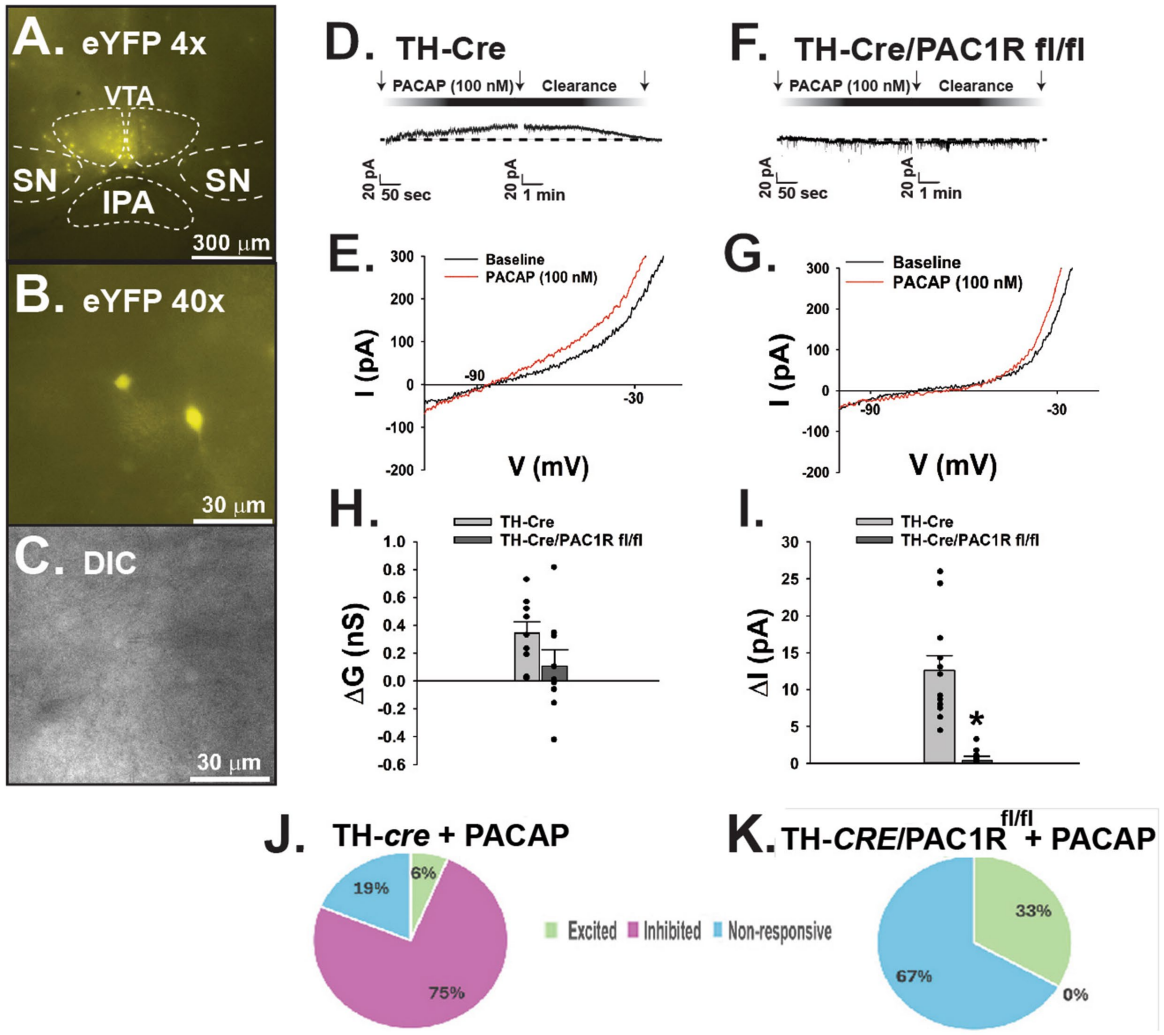
Lack of PAC1R attenuates PACAP induced outward current in VTA A_10_ DA neurons in TH-*cre*/PAC1R^fl/fl^ mice. **(A)** Low powered (4X) photomicrograph of eYFP-fluorescing A_10_ DA neurons localized to the VTA. **(B)** High powered (40X) photomicrograph illustrating a representative A_10_ DA neuron about to undergo electrophysiologic recording. **(C)** Corresponding DIC image of the recorded TH neuron. **(D)** Representative outward current elicited in A_10_ DA neurons in TH-*cre* mice upon bath application of PACAP_1-38_. **(E)** I/V plot showing a −90 mV reversal potential corresponding with the Nernst equilibrium potential for potassium conductance. **(F)** Representative voltage clamp trace depicting no significant change in membrane current in A_10_ DA neurons recorded from TH-*cre*/PAC1R^fl/fl^ animals. **(G)** I/V relationship showing no significant change in slope conductance compared to baseline. **(H,I)** Composite data depicting slope conductance and membrane current are lower in TH-*cre*/PAC1R^fl/fl^ animals following bath application of PACAP. Student’s *t*-test, ^*^p < 0.05, TH-*cre n* = 6 animals, 9 slices, 9 cells, TH-*cre*/PAC1R^fl/fl^ *n* = 5 animals, 5 slices, 9 cells. Bars represent means and lines 1 SEM. **(J,K)** Pie charts reflecting the distribution of A_10_ dopamine neurons from TH-*cre* and TH-*cre*/PAC1Rfl/fl animals that are inhibited, excited or unresponsive to PACAP.

Following establishment of the PAC1R knockdown in A_10_ DA neurons, we tested whether PACAP_1-38_ would still induce hyperpolarization of these cells in the absence of the PAC1R. We conducted whole-cell patch clamp recordings of A_10_ DA neurons visualized with eYFP (Figures 2A–C) in both TH-*cre* and TH-*cre*/PAC1R^fl/fl^ mice. The representative traces and I/V plots reveal that bath application of PACAP_1–38_ (100 mM) induced a robust outward current in A_10_ DA neurons from TH-*cre* mice (Figure 2D), with a corresponding increase in slope conductance and reversal potential of −90 mV (Figure 2E). In contrast, bath application of PACAP_1–38_ had no significant effect on membrane current from recorded A_10_ DA neurons nor a change in slope conductance in TH-*cre*/PAC1R^fl/fl^ mice (Figures 2F,G). These are largely corroborated when looking at the composite data, with the slope conductance (ΔG) reduced by 69% (Figure 2H: student’s *t*-test, *t* = 1.649, DF: 16, *p* < 0.12) and the membrane current (Figure 2I: student’s *t*-test, *t* = 5.160, DF: 16, *p* < 0.0001) negligible in TH-*cre*/PAC1R^fl/fl^ compared to TH-*cre* mice with intact PAC1Rs. These data suggest the PAC1R is necessary to effectuate the inhibitory effect of PACAP on VTA A_10_ DA neurons (Figures 2J,K).

### 3.3 PAC1R knockdown abrogates the intra-VTA PACAP-induced decrease in binge intake, frequency, and bout duration in TH-*cre* mice

**Figure 3:**
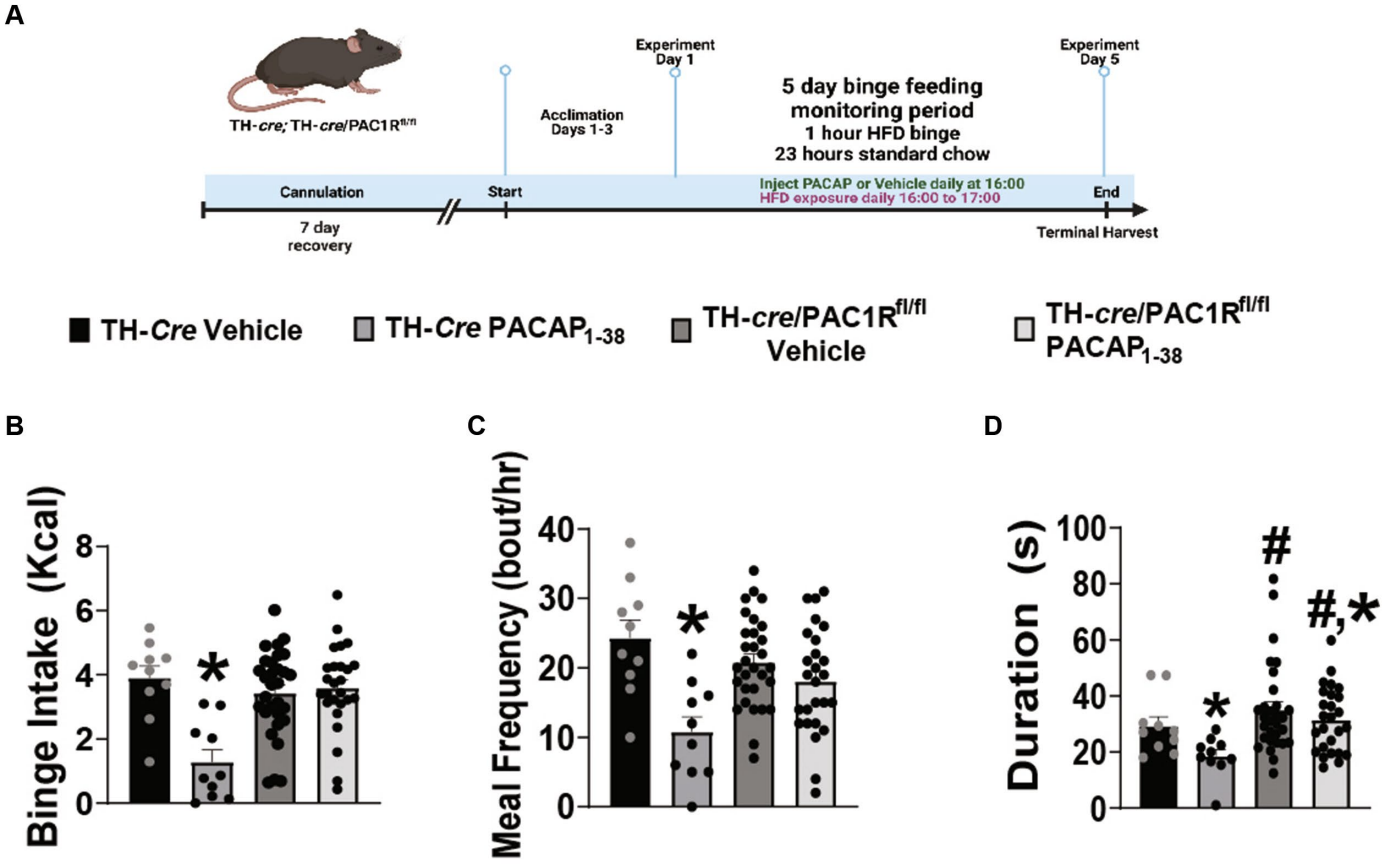
Intra-VTA PACAP_1-38_ significantly reduces binge intake, meal frequency, and bout duration in TH-*cre* but not TH-*cre*/PAC1R^fl/fl^ mice. **(A)** Schematic illustrating the procedural timeline for the execution of these experiments. **(B–D)** Composite data depicting PACAP_1–38_ significantly decreases binge intake **(B)**, meal frequency **(C)**, and bout duration **(D)** in TH-*cre* mice, effects which are largely negated in the absence of PAC1R in VTA TH neurons. ^*^p < 0.05 relative to vehicle, ^#^*p* < 0.5 relative to genotype. Repeated measures, multifactorial ANOVA/LSD, TH-*cre* vehicle *n* = 10, TH-*cre* PACAP_1–38_ *n* = 9, TH-*cre*/PAC1R^fl/fl^ vehicle *n* = 30, and TH-*cre*/PAC1R^fl/fl^ *n* = 26. Bars represent means and lines 1 SEM.

Seeing as how exogenous PACAP elicited no significant effect on recorded A_10_ DA neurons from TH-*cre/*PAC1R^fl/fl^ mice, we therefore postulated that knockdown of the PAC1R would also negate the PACAP-induced inhibition of binge-like behavior. TH-*cre* and TH-*cre*/PAC1R^fl/fl^ groups both were surgically outfitted with a guide cannula situated just above the VTA to allow for focal injection of PACAP_1–38_ (30 pmol) or its saline vehicle (0.9%) and underwent intermittent 1-h exposure to HFD as previously described and validated (Figure 3A) (38). Pre-surgical weights were not significantly different between the two groups (TH-*cre*—23.8 ± 0.6 g, TH-*cre*/PAC1R^fl/fl^—24.8 ± 1.6 g student’s *t*-test, *t* = 0.6705, DF: 84, *p* < 0.51). Intra-VTA administration of PACAP_1–38_ significantly just prior to this HFD exposure decreased binge intake in TH-*cre* mice (Figure 3B). This PACAP-induced decrement is associated with a reduction in both meal frequency (Figure 3C) and bout duration (Figure 3D) in TH-*cre* but not TH-*cre*/PAC1R^fl/fl^ mice. The decreases in binge intake (Figure 3B) and meal frequency (Figure 3C) caused by PACAP were largely abolished in TH-*cre*/PAC1R^fl/fl^ mice (Figure 3B: repeated measures multi-factorial ANOVA/LSD: *F*_PACAP1-38_: 13.42, DF: 1, *p* < 0.0006; *F*_genotype_: 5.86, DF: 1, *p* < 0.02; *F*_interaction:_ 13.94, DF: 1, *p* < 0.0005, one-way ANOVA/LSD: *F* = 8.12, *p* < 0.0002; Figure 3C: repeated measures multi-factorial ANOVA/LSD: *F*_PACAP1-38_: 19.19, DF: 1, *p* < 0.0001; *F*_genotype_: 0.63, DF: 1, *p* < 0.43; *F*_interaction:_ 6.79, DF: 1, *p* < 0.02, one-way ANOVA/LSD: *F* = 6.74, *p* < 0.0005). There was a significant main effect of both PACAP_1–38_ and genotype on bout duration, but no interaction between the two factors, and we interpreted this to mean that the PACAP-induced diminution of this meal pattern index was attenuated in TH-*cre*/PAC1R^fl/fl^ mice (Figure 3D: repeated measures multi-factorial ANOVA/LSD: *F*_PACAP1-38_: 4.38, DF: 1, *p* < 0.04; *F*_genotype_: 7.58, DF: 1, *p* < 0.008; *F*_interaction:_ 1.11, DF: 1, *p* < 0.30). Thus, the knockdown of the PAC1R in A_10_ DA neurons greatly compromises the inhibitory effect of intra-VTA PACAP on binge feeding behavior.

### 3.4 Apoptotic lesioning of VMN PACAP neurons enhances hedonic drive in PACAP-*cre* animals

**Figure 4:**
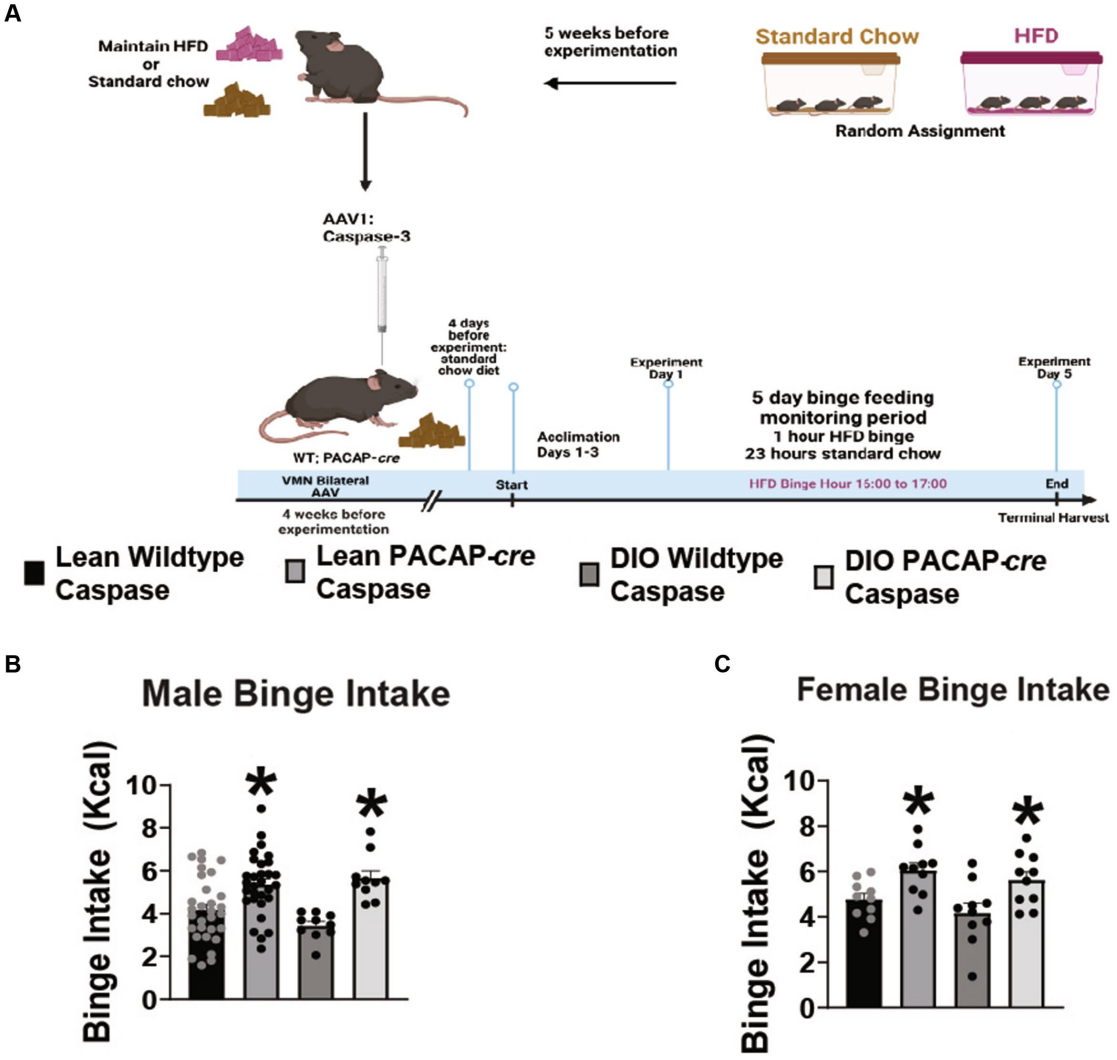
VMN PACAP neuron ablation in PACAP-*cre* mice increases binge consumption in males and females. **(A)** Schematic illustrating the procedural timeline for the execution of these experiments. **(B,C)** Composite data depicting caspase ablation of VMN PACAP neurons in PACAP-cre mice elicits a significant increase in binge consumption in both lean and obese male **(B)** as well as female **(C)** mice. ^*^p < 0.05 relative to wildtype controls. Repeated measures, multifactorial ANOVA/LSD, **(B)** lean wildtype *n* = 30, lean PACAP-*cre n* = 30, DIO wildtype *n* = 10, DIO PACAP-*cre n* = 10; **(C)** lean wildtype *n* = 10, lean PACAP-*cre n* = 10, DIO wildtype *n* = 10, DIO PACAP-*cre n* = 10. Bars represent means and lines 1 SEM.

Because VMN PACAP neurons project to and terminate in the VTA, and optogenetic stimulation of these cells inhibits A_10_ DA neurons (35), we tested the hypothesis that apoptotic ablation of VMN PACAP neurons would enhance hedonic drive. Lean, chowfed, and DIO HFD-fed male and female wildtype and PACAP-*cre* cohorts were injected with caspase-3 AAV in the VMN to apoptotically lesion VMN PACAP neurons 4 weeks prior to experimentation as described and validated previously (Figure 4A)(23). Caspase ablation promoted a significant increase in binge consumption in both lean and obese male (Figure 4B: repeated measures multi-factorial ANOVA/LSD: *F*_genotype_: 23.88, DF: 1, *p* < 0.0001; *F*_diet_: 0.38, DF: 1, *p* < 0.55; *F*_interaction_: 2.15, DF: 1, *p* < 0.15) and female (Figure 4C: repeated measures multi-factorial ANOVA/LSD: *F*_genotype_: 15.01, DF: 1, *p* < 0.0005; *F*_diet_: 2.06, DF: 1, *p* < 0.16; *F*_interaction_ genotype/diet: 0.07, DF: 1, *p* < 0.80) subjects. Thus, VMN PACAP neurons may tonically inhibit the hedonic consumption of palatable food irrespectively of sex or energy status. In comparing body weight of TH-*cre* and TH-*cre*/PAC1R^fl/fl^ animals utilized for both feeding and electrophysiology studies, starting weight was not significantly different (student’s *t*-test, *t* = 0.6705, DF: 84, *p* > 0.05).

## 4 Discussion

The data curated throughout this project indicate PACAP-induced inhibition of VTA A_10_ DA neurons is PAC1R-dependent. The PAC1R is necessary for PACAP’s ability to exert its inhibitory effects on VTA A_10_ DA neurons, and attenuation of the PACAP mediated inhibition upon knockdown of the PAC1R in VTA A_10_ DA neurons increases hedonic drive. Additionally, VMN PACAP neurons tonically reduce hedonic drive independent of sex and diet. We have confirmed exogenous PACAP inhibits of VTA A_10_ DA neurons as evidenced by the robust and reversible outward current bath application of PACAP_1-38_ promoted in recordings of VTA A_10_ DA neurons. Following knockdown of the PAC1R in these A_10_ DA neurons, bath application of PACAP_1-38_ elicited no significant change in A_10_ DA membrane current. The reduced PACAP-mediated inhibition of A_10_ DA neurons greatly attenuates the decreased binge intake as well as meal frequency and bout duration during intermittent exposure to highly palatable food. Lastly, ablation of VMN PACAP neurons promoted increased drive for highly palatable food consumption, in both male and female groups, as well as lean and DIO sub-groups, confirming that VMN PACAP neurons tonically inhibit VTA A_10_ DA neurons.

### 4.1 Knockdown of PAC1R functionality in VTA A_10_ DA neurons diminishes PACAP induced inhibition

Retrograde tracing has indicated VMN PACAP neurons project to and terminate at VTA A_10_ DA neurons (35). Optogenetic stimulation of VMN PACAP neurons inhibits VTA neurons. This inhibitory effect was replicated with exogenous bath application of PACAP_1–38_ in VTA A_10_ DA neurons and diminished in the presence of K_ATP_ channel blocker and PAC1R antagonist, tolbutamide and PACAP_6-38_, respectively (35). PACAP selectively binds to PAC1R with high affinity, however, this neuropeptide also binds to VPAC1R and VPAC2R (39). It has been shown that the PAC1R/VPAC2R antagonist PACAP_6-38_ abrogates the appetite-suppressing effects of PACAP in several different species, including goldfish and chicks; indicating PACAP’s anorexic effects are PAC1R mediated (40, 41). In the present study, we show knockdown of the PAC1R in VTA A_10_ DA neurons renders the bath application of exogenous PACAP in mesencephalic slices ultimately ineffective. This was evidenced by the significant attenuation in change in A_10_ DA membrane current, as well as a lesser increase in slope conductance. These data confirm and extend PAC1R’s role in mediating PACAP induced inhibition of A_10_ DA neurons.

### 4.2 Blunted PACAP-mediated inhibition of VTA A_10_ DA neurons increases tendencies toward binge feeding behavior during intermittent exposure to highly palatable food

The NAc has been strongly tied to appetitive motivation in both rodents and humans (42–44). The incentive-sensitization model of obesity suggests hyper-responsivity of the DA reward circuitry is due to repeated pairings of reward from food intake and food-intake associated cues (45). In accordance with this theory, the incentive-sensitization model of drug addiction asserts that hypersensitivity to motivational stimuli associated with high incentive salience promotes attentional processing bias toward reward-related cues (46). This bias toward reward-related cues is proposed to trigger the release of DA and drive consumption (47). The same underlying processes most likely extend to food addiction and eating disorders like bulimia. Indeed, our binge feeding paradigm results in a dramatic escalation in the ingestion of palatable food over the 5 days of the monitoring period; resulting in upwards of 40% of the daily caloric intake being consumed during the one-hour HFD exposure. This escalation is blunted by PACAP and other neuropeptides like nociceptin/orphanin FQ due to their ability to inhibit A_10_ dopamine neurons (35, 38).

Food cues act as stimuli, which promote the urge to eat. This is evidenced by rodent studies depicting increased cue-triggered motivation and a sensitized mesolimbic system following high fat diet (HFD) consumption resulted in increased activity of DA cells and increased expression of the rate limiting enzyme of DA synthesis, tyrosine hydroxylase (TH), within the NAc (48). Studies with clinically obese populations have reported stronger cue triggered food cravings coupled with larger portion consumption (47). Additionally, this study demonstrated that body mass index (BMI) is positively correlated with food-seeking behavioral responses, which implied food cues triggered a greater attention bias toward food in overweight versus lean individuals (47).

Pituitary adenylate cyclase-activating polypeptide and its cognate receptors are expressed in the NAc and VTA, which are evidenced to be involved in the hedonic regulation of feeding (5, 33, 34). In accordance with this, PACAP administered to the NAc reduced hedonic feeding without affecting homeostatic feeding (31, 32). Likewise, intra-VTA PACAP administration suppresses the binge-like consumption of palatable food in lean mice due to activation of K_ATP_ channels and hyperpolarization of A_10_ DA neurons in the VTA (35), which is blocked by the PAC1/VPAC2R antagonist PACAP_6–38_. In the present study, intra-VTA administration of PACAP_1–38_ significantly decreased binge intake, meal frequency, and bout duration during intermittent exposure to HFD in TH-*cre*, but not TH-*cre/*PAC1R^fl/fl^ animals. The most parsimonious explanation of this finding is that knockdown of the PAC1R attenuated the suppressive effect promoted by PACAP on hedonic feeding. TH-*cre/*PAC1R^fl/fl^ animals exhibited binge feeding behavior virtually indistinguishable from their TH-*cre* saline vehicle-treated counterparts despite receiving intra-VTA administration of PACAP_1–38_. Therefore, the reduced PACAP mediated inhibition of A_10_ DA neurons in TH-*cre*/PAC1R^fl/fl^ animals exhibited in our electrophysiology studies translates behaviorally to decreased inhibition of the hedonic feeding behavior. Admittedly, we did not see an elevation in binge feeding *per se* in the vehicle treated, TH-*cre/*PAC1R^fl/fl^ animals, which suggests that activation of PAC1Rs by PACAP do not tonically inhibit hedonic feeding. We also reported no significant difference in starting body weight for TH-*cre* and TH-*cre/*PAC1R^fl/fl^ animals. While this data was solely from pre-surgical body weight, previous research followed body weight across an eight-week timespan and reported a significant increase in body weight gain in caspase induced ablation of VMN PACAP neurons (23). Additionally, this research depicted PACAP inhibits AgRP neurons resulting in decreased energy intake and expenditure, however, upon knockdown of the PAC1R in AgRP neurons, there was no significant difference in energy intake nor expenditure with application of PACAP. Nevertheless, our findings demonstrate the PAC1R can play a role in inhibition of the hedonic mesolimbic pathway. PACAP activation of the PAC1R, and subsequent activation of K_ATP_ channels (5), decreases A_10_ DA activity; desensitizing the mesolimbic system to food cues and decreasing hedonic drive toward highly palatable food.

### 4.3 VMN PACAP neurons are a key proponent for inhibition of the mesolimbic DA pathway and attenuation of hedonic behavior

The *cre*-dependent apoptotic ablation of VMN PACAP neurons promoted a significant increase in hedonic feeding behavior in both male and female, lean and obese cohorts. The significant increase in hedonic drive strongly indicates that VMN PACAP neurons are necessary for the tonic inhibition of VTA A_10_ DA neurons. In previous studies, we have demonstrated that optogenetic stimulation of VMN PACAP neurons effectively inhibit VTA neurons (35). VMN PACAP neurons contain other important phenotypic markers including glutamate steroidogenic factor-1 (24, 49–51). Considering that acute knockdown of PAC1R in A_10_ dopamine neurons *per se* did not affect binge feeding, it is entirely plausible that glutamate released from these neurons carry out this tonic inhibition via activation of metabotropic glutamate receptor, and future studies will determine if this is in fact the case. Nevertheless, it is apparent that VMN PACAP neurons play a key role in the inhibition of VTA A_10_ DA neurons and consequently the reduction in drive for hedonic feeding. On the other hand, the PACAP-induced diminution of binge feeding behavior is sex dependent in that intra-VTA PACAP decreases binge feeding in lean, otherwise chow-fed males but not in their female counterparts (35). Moreover, PACAP delivery into the VTA of obese females actually increases binge feeding behavior (5). Studies have shown that women have a greater risk of developing food addiction. This may be due, in part, to greater activation of the dorsolateral prefrontal cortex (dlPFC), medial orbitofrontal cortex, and the amygdala when presented with a food cue (52). Activation of the dlPFC is increased during reward anticipation, and this enhanced activity of the dlPFC stimulates A_10_ DA neurons (53). The medial orbitofrontal cortex and amygdala receive A_10_ DA input and are important for goal directed behavior and processing of emotional stimuli associated with food cues (54, 55). Additionally, binge studies in female rats depicted heightened tolerance to high levels of foot shock in acquiring Oreo cookies—indicating a food addiction defined as sustained desire for food consumption despite negative consequences (56, 57). Within the mesolimbic pathway, estradiol regulates dopamine neuron sensitization in females (58, 59), an effect that is not present in males (60). It follows then that estradiol’s role in sensitization is a likely underlying factor in the sexually differentiated disparities in addiction and hedonic feeding behavior. However, in the present study we found that apoptotic ablation of VMN PACAP neurons increased binge feeding behavior in both lean and obese, male and female PACAP-*cre* animals. As mentioned above, VMN PACAP neurons are glutamatergic (24, 50, 51), and it bears repeating that glutamate acting at inhibitory metabotropic glutamate receptors may very well be responsible for the tonic suppression of binge feeding that is relieved upon apoptotic ablation of these cells. On the other hand, the actions of PACAP in the VTA with respect to hedonic feeding are clearly sexually differentiated; as PACAP decreases binge feeding in lean males but not females and increases it in obese females (5, 35). Thus, once sex and energy status have been factored in appropriately. The PACAP/PAC1R system may very well prove to be an effective target to curb excessive eating via inhibiting the hedonic feeding pathway.

In conclusion, we have demonstrated that the PACAP-induced inhibition of the mesolimbic pathway is reliant on PAC1R activation. Taken together, these data demonstrate the contribution of the PACAP/PAC1R system and VMN PACAP neurons in reducing excitability of the mesolimbic dopamine pathway via inhibition of VTA A_10_ DA neurons that in turn suppresses hedonic feeding. This knowledge renders the PAC1R a potential therapeutic target in managing excessive eating.

## Data Availability

The raw data supporting the conclusions of this article will be made available by the authors, without undue reservation.
